# Effect of Mn/Ag Ratio on Microstructure and Mechanical Properties of Heat-Resistant Al-Cu Alloys

**DOI:** 10.3390/ma17061371

**Published:** 2024-03-17

**Authors:** Xiangzhou Fu, Hailong Yang, Hanzhang Wang, Chifu Huang, Yongbin Chen, Qiangang Huang, Anmin Li, Liwen Pan

**Affiliations:** 1 School of Resources, Environment and Materials, Guangxi University, Nanning 530004, China; fxz19990720@163.com (X.F.); 18743203440@163.com (H.Y.); whz1015167594@163.com (H.W.); hcf1094099919@163.com (C.H.); 19175363571@163.com (Y.C.); 18778610879@163.com (Q.H.); lamanny@126.com (A.L.); 2 State Key Laboratory of Featured Metal Materials and Life-Cycle Safety for Composite Structures, Nanning 530004, China; 3 Key Laboratory of High Performance Structural Materials and Thermo-Surface Processing, Education Department of Guangxi Zhuang Autonomous Region, Guangxi University, Nanning 530004, China

**Keywords:** Al-Cu-Mn-Ag alloy, Mn/Ag ratio, microstructure, high-temperature tensile mechanical property

## Abstract

This paper mainly investigated the effect of the Mn/Ag ratio on the microstructure and room temperature and high-temperature (350 °C) tensile mechanical properties of the as-cast and heat-treated Al-6Cu-xMn-yAg (x + y = 0.8, wt.%) alloys. The as-cast alloy has α-Al, Al_2_Cu, and a small amount of Al_7_Cu_2_ (Fe, Mn) and Al_20_Cu_2_ (Mn, Fe)_3_ phases. After T6 heat treatment, a massive dispersive and fine θ′-Al_2_Cu phase (100~400 nm) is precipitated from the matrix. The Mn/Ag ratio influences the quantity and size of the precipitates; when the Mn/Ag ratio is 1:1, the θ′-Al_2_Cu precipitation quantity reaches the highest and smallest. Compared with the as-cast alloy, the tensile strength of the heat-treated alloy at room temperature and high temperature is greatly improved. The strengthening effect of the alloy is mainly attributed to the nanoparticles precipitated from the matrix. The Mn/Ag ratio also affects the high-temperature tensile mechanical properties of the alloy. The high-temperature tensile strength of the alloy with a 1:1 Mn/Ag ratio is the highest, reaching 135.89 MPa, 42.95% higher than that of the as-cast alloy. The analysis shows that a synergistic effect between Mn and Ag elements can promote the precipitation and refinement of the θ′-Al_2_Cu phase, and there is an optimal ratio (1:1) that obtains the lowest interfacial energy for co-segregation of Mn and Ag at the θ′/Al interface that makes θ′-Al_2_Cu have the best resistance to coarsening.

## 1. Introduction

Heat-resistant aluminum alloy refers to a light alloy with high strength, good oxidation resistance, good creep resistance, good fatigue resistance, and good thermal conductivity at high temperatures. Moreover, the manufacturing cost is relatively low. Therefore, it is widely used in automobiles, aerospace technology, ships, and weapons [1,2,3,4,5].

The heat resistance of cast Al-Cu alloy is the best among the heat-resistant aluminum alloys. It has good mechanical properties at room temperature and high temperatures after heat treatment. The high-temperature strengthening effect of cast Al-Cu alloy is mainly attributed to the precipitated θ′-Al_2_Cu phase after solution–aging treatment. After solution–aging, the fine and dispersed θ′-Al_2_Cu metastable phase precipitates in the Al-Cu alloy matrix, which has a semi-coherent relationship with the matrix and can effectively block dislocation movement and pin grain boundaries, thus improving the high-temperature strength and creep resistance of the alloy [6,7]. For Al-Cu alloys, the rich microstructure of θ′-Al_2_Cu provides the best strength and thermal stability for high-temperature applications [8,9,10,11,12,13,14]. θ′-Al_2_Cu has a four-angle distorted fluorite structure (space group I4/MMM) where a = 0.404 nm and C = 0.580 nm. In the FCC Al-rich matrix with wide (001) common-lattice and semi-common-lattice interfaces, θ′-Al_2_Cu precipitates as a plate on a {001} surface, and the plate (Al_2_Cu) precipitation is one of the main strengthening phases of Al-Cu alloys. With the development of science and technology, the service temperature of high-power engines, high-speed aircraft skins, and other devices has come to be 300~400 °C. However, the use temperature of traditional cast heat-resistant Al-Cu alloys has reached the limit state, unable to meet the heat resistance requirements of these devices. The main reason is that the thermal stability of the main strengthening phase θ′-Al_2_Cu in Al-Cu alloys is only about 225 °C, and above 250 °C, it is easy to transform to the coarser θ-Al_2_Cu and the strengthening effect is significantly decreased, so the use temperature of the general heat-resistant Al-Cu alloy does not exceed 225 °C [15]. In recent years, further improving the heat resistance of Al-Cu alloys by introducing high thermal stability strengthening phases or increasing the thermal stability of the θ′-Al_2_Cu phase with microalloying has become a research hotspot in this field.

Mn is a common element in aluminium alloys [16,17,18,19,20]. It is found that adding trace Mn to the alloy can improve the microstructure of the alloy, reduce the harm of the impurity element Fe, form the thermal stability strengthening phase T (Al_20_Cu_2_Mn_3_) in the alloy, and improve the heat resistance of the alloy [21]. The diffusion rate of the Mn atom is slower than that of the Cu atom, and the presence of Mn can slow down the diffusion rate of Cu at high temperatures. Mn elements generally tend to segregate at the interface of θ′-Al_2_Cu/α-Al, reducing the interfacial energy of the alloy, thereby reducing the driving force of θ′-Al_2_Cu phase growth, refining the θ′-Al_2_Cu phase and improving the thermal stability of the precipitated phase [22,23,24,25]. In recent years, significant progress has been made in studying the segregation behavior and thermal stability of the θ′/Al interface by adding Mn in combination with other elements. Jonathan D. Poplawsky et al. [25] studied the segregation behavior of Mn and Zr/Ti at the metastable θ′ interface in Al-Cu alloys using APT and STEM. The results show that jointly adding Mn and Zr can increase the thermal stability of θ′ to 350 °C. Adding Zr and Mn alone can only increase the thermal stability of the θ′ phase to 200 °C and 300 °C, respectively. This shows that the two have the function of synergistically stabilizing the θ′ phase. This analysis showed that the addition of Mn stabilized θ′ for a long enough time so that Zr atoms with slower diffusion rates were segregated to the θ′ interface and finally formed a θ′/L1_2_-Al_3_ (Zr_x_, Ti_1−x_) co-precipitation structure, which was the reason for significantly improving the thermal stability of θ′. Lu Jiang et al. [26] investigated the effects of the coupled segregation mechanism of Sc, Zr, and Mn at the θ′ interface on the Al-Cu alloy’s strength and thermal stability and found that adding Sc and Zr could mainly refine the θ′ phase. In contrast, Mn not only significantly refined the θ′ phase but also significantly improved the anti-coarsening ability of the θ′ phase and improved the alloy’s hardness after aging. The calculations showed that the segregation of Mn at the θ′ interface dramatically reduced the interface energy and provided a kinetic diffusion barrier through solute drag, thereby reducing the nuclear barrier and improving the anti-coursing ability of the θ′ phase. Guangjing Li et al. [27] studied the effect of Mn on the Al-Cu alloy’s microstructural evolution and high-temperature strength. They found that during long-term thermal exposure, Mn atoms would continue to enrich in front of the growth interface of the θ′ phase, hindering their coarsening and thus inhibiting the θ′ to θ phase transition. After treating the alloy at 300 °C for 100 h, they found that the yield strength of the Al-Cu alloy increased by 15% at 300 °C after adding the Mn element. However, this still could not meet the current requirements for the heat resistance of aluminium alloy, and the heat resistance of Al-Cu-Mn alloy needs to be improved.

In aluminium alloys, Ag is often introduced into Al-Cu alloys with a high Cu/Mg ratio, where Ag and Mg combine to form Mg-Ag atomic clusters, which provide a location for the nucleation of a new phase (Ω) and significantly increase the number of the Ω precipitates [28,29]. Ω phase is a distorted form of the θ′ phase and is semi-coherent with the Al matrix. Compared with θ′, the Ω phase has high thermal stability, so it has good resistance to coarsening [30,31]. Compared with traditional aluminium alloys such as 2219 and 2618, Al-Cu-Mg alloys containing Ag have excellent mechanical properties at higher temperatures, such as tensile strength and creep resistance [32,33,34,35,36,37,38]. Chen et al. [39] optimized the heat treatment process of an Al-Cu-Mg-Ag alloy and studied the effect of different annealing temperatures on the high-temperature mechanical properties of the alloy. They obtained a tensile strength of 301.9 MPa at 250 °C. However, the research on the heat resistance of Al-Cu-Mg-Ag alloys is still limited, and research on the mechanical properties at higher temperatures is rarely reported. Julian M. Rosalie et al. [40] found that Ag segregated to the θ′/α-Al coherent interface in an Al-Cu-Ag alloy. Ag segregated to approximately 40% of the semi-coherent θ′-Al_2_Cu/α-Al interfaces, which acted as a barrier to Cu diffusion, thus impeding or preventing lateral growth of the θ′ precipitates. However, this study stopped at the characterization of the alloy’s microstructure, and the factors affecting the mechanical properties of the alloy are more complex, which is worthy of further verification by practical experimental methods.

Numerous studies have confirmed the synergistic effect of Ag and Mg in Al-Cu alloys [28,30,31,41,42,43], promoting the precipitation and refinement of the Ω phase and improving the high-temperature mechanical properties of the alloy. However, few reports exist on the synergistic modification effect of Ag and other elements in Al-Cu alloys. The modification effect of Ag alone and Mn alone on Al-Cu alloys is similar. However, it has yet to be reported whether there is a synergistic effect when both are simultaneously added to an Al-Cu alloy. This research mainly studied the changes in the microstructure, room temperature, and high-temperature mechanical properties of an Al-Cu alloy after the simultaneous addition of Mn and Ag. It explored the synergistic modification mechanism of the two elements, laying a foundation for developing new heat-resistant aluminium alloys.

## 2. Materials and Methods

The primary materials used in this experiment included industrial pure aluminium (99.7 wt.%), an Al-50Cu master alloy, an Al-10Mn master alloy, and pure silver powder (99.99 wt.%), and the nominal components of the alloys are shown in Table 1. The first five groups were experimental groups to explore the effect of changing the Mn/Ag ratio on the mechanical properties and microstructures of the alloy. We then added a control group of Al-6Cu without Mn or Ag elements. In the experiment, a coating agent (50 wt.% NaCl + 50 wt.% KCl), refining agent (C_2_Cl_6_ powder), and crucible coating (Na_2_SiO_3_:ZnO = 1:3, wt.%) were also used. These reagents are from Xiongrun Chemical, Nanning, China.

Firstly, the dried aluminium ingot was placed into the graphite crucible, which was installed in the coil of a medium-frequency induction furnace (Yihui Castino, Guangzhou, China). Then, we turned the power on and the heat up. After the aluminium was completely melted, the melt surface was covered with a coating agent to form a continuous covering layer, which prevented the oxidation and volatilization of the aluminium liquid. During the continuous heating process of the melt, the thermocouple end of a handheld thermometer was inserted into the melt to measure the melt temperature. The melt temperature was controlled at about 760~770 °C for 6–7 min by adjusting the heating power. Subsequently, slagging, Al-Cu, and the Al-Mn master alloy were added to the melt, and the melt temperature was held for 8 min. After slagging again, the silver powder was added, and the melt was mechanically stirred at 300 r/min for 2 min to make the silver powder entirely diffused. Then, the rotating speed was increased to 600 r/min for 2 min. After stopping the stirring, we let the melt stand for 5 min and cool down to around 740 °C. After slagging again, two degassing operations were performed on the melt using hexachloroethane and manual stirring. Finally, we let the melt stand for 3 min and cool down to about 720 °C, slagged it again, and cast it into the mold preheated at 300 °C. The sample was demolded after the mold was cooled down to room temperature.

According to some reports [44], when the addition of the Ag element in an Al-Cu alloy reaches a certain amount, the precipitation of the Al_2_Cu phase will no longer have a significant impact, and its content generally would not exceed 0.6 wt.%. There is also a suitable range for the amount of Mn added. When the Mn content is too high, the insoluble T phase in the structure of an Al-Cu alloy increases, forming a continuous network, which will split the matrix, make the alloy brittle, and reduce the tensile strength of the alloy at room temperature. Referring to some other reports [15], the Mn content was generally below 0.8 wt.%. Therefore, the total content of the Mn and Ag elements was set as 0.8 wt.% in this experiment, and the synergistic effect between the Mn and Ag elements in the Al-Cu alloy was explored by changing the Mn/Ag ratio.

**Figure 1:**
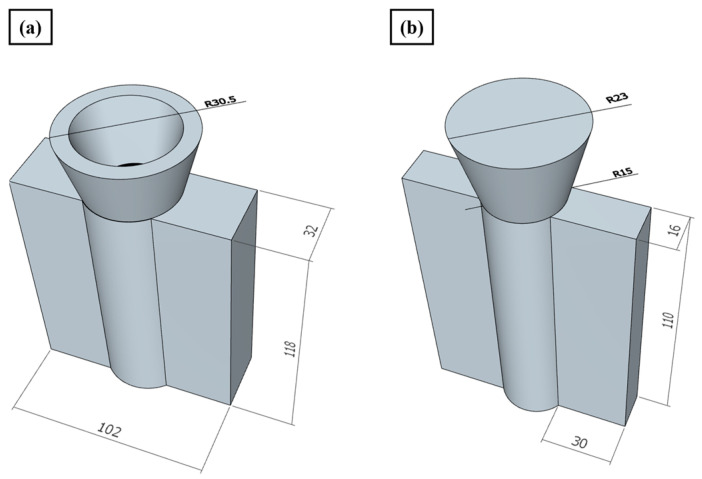
Schematic diagrams of the steel mold and ingot casting: (**a**) steel mold; (**b**) ingot casting.

**Figure 2:**
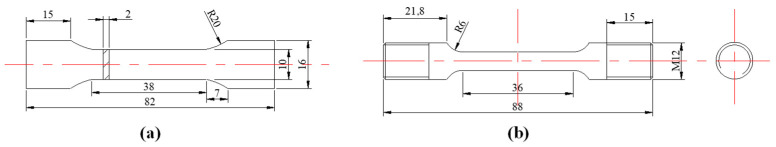
Dimensions and of the tensile specimens: (**a**) room temperature tensile sample; (**b**) high-temperature (350 °C) tensile sample.

We cast the metal liquid into a mold made of 1045 steel (shown in Figure 1), waited for the casting to cool to room temperature, and removed the mold. The casting was divided into as-cast and heat-treated samples. The as-cast sample was divided into microstructure observation, room temperature, and high-temperature tensile samples. The heat-treated sample was first subjected to T6 heat treatment and then divided into microstructure observation, room temperature, and high-temperature tensile samples. The room temperature and high-temperature tensile samples were processed by wire cutting technology, and the specific dimensions are shown in Figure 2.

The matrix alloys were subjected to T6 heat treatment to precipitate the dispersion θ′-Al_2_Cu-strengthening phase from the matrix. When the temperature reached 300 °C, the prepared Al-Cu-Mn-Ag alloy was placed in the box resistance furnace (Shiyan electric furnace, Shanghai, China) for annealing treatment and held for two hours. After the end of the heat preservation, it was cooled with the furnace. Referring to the previous heat treatment process [45,46], we carried out the solid solution and aging heat treatment of the alloy in a box-type resistance furnace: 540 °C/12 h + 25 °C/WC + 170 °C/8 h/AC.

A PhenomProX-type integrated scanning electron microscope (SEM) and an energy dispersive spectrometer (EDS) (Phenom World, Eindhoven, The Netherlands) were used to observe and analyze the surface morphology and phase composition of the metallographic samples and the tensile fracture samples.

MiniFlex600 physical X-ray diffraction (XRD) (Rigaku Corporation, Tokyo, Japan) was used to analyze the phase of the alloy samples. The scanning speed was 6°/min, and the scanning angle range was 20°~80°. The obtained XRD diffraction pattern was compared with the PDF card to determine the phase corresponding to each diffraction peak. Room temperature and high-temperature tensile tests were carried out with a BWN100KN series desktop computer control electronic universal testing machine (Airma, Shenzhen, China) with a high-temperature heating system. The tensile rate was 0.5 mm/min. The high-temperature tensile temperature was set to 350 °C, and the control temperature accuracy was ±1 °C. Before the high-temperature tensile test, the sample was held at 350 °C for 20 min.

## 3. Results and Discussion

### 3.1. Microstructure Analysis

**Figure 3:**
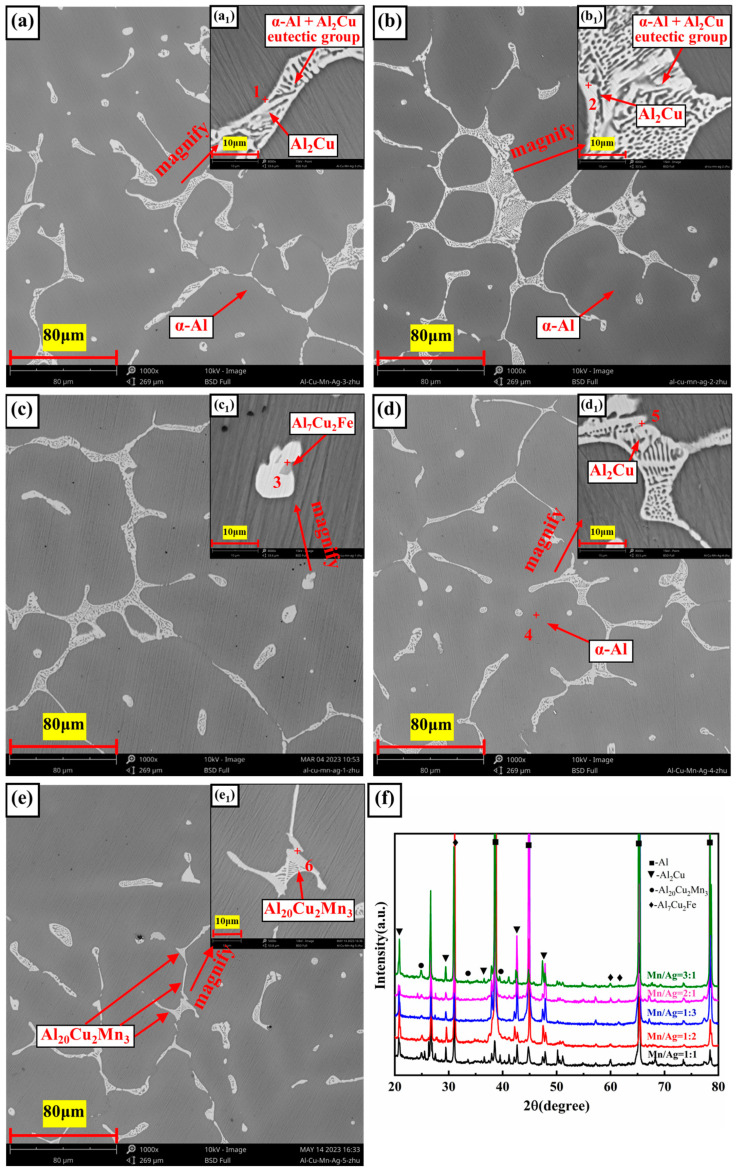
Microstructure of the as-cast Al-6Cu-xMn-yAg alloys: (**a**,**a_1_**) x/y = 1:3; (**b**,**b_1_**) x/y = 1:2; (**c**,**c_1_**) x/y = 1:1; (**d**,**d_1_**) x/y = 2:1; (**e**,**e_1_**) x/y = 3:1; (**f**) XRD patterns of as-cast alloys.

**Figure 4:**
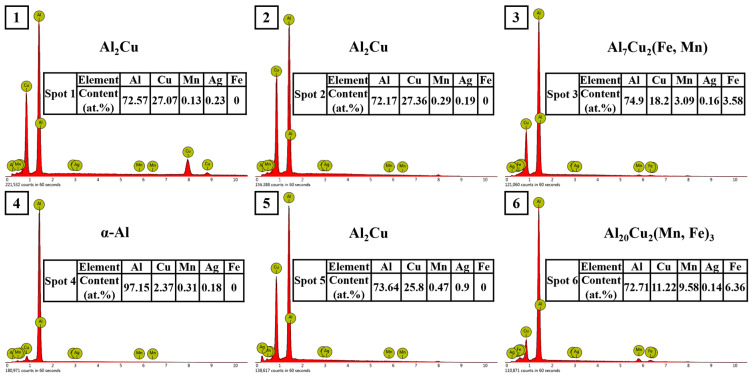
Energy spectrogram of composition at each point in Figure 3.

The SEM micrographs and X-ray diffraction (XRD) images of the Al-6Cu-xMn-yAg alloy are shown in Figure 3. The chemical composition and energy dispersion spectrum (EDS) analyses of each phase of the alloy are shown in Figure 4. As shown in Figure 4, the as-cast microstructure of the alloy was mainly composed of α-Al, Al_2_Cu, Al_20_Cu_2_Mn_3_, and Al_7_Cu_2_Fe phases.

The dark gray α-Al (spot 4) was the matrix phase with a dendritic structure. We performed EDS analyses on the bright white reticular phase in the image (spots 1, 2, and 5), and the results showed that this phase was mainly composed of Al and Cu elements, and the atomic ratio of the Al and Cu elements was close to 2:1. Combined with the obvious Al_2_Cu diffraction peak found in the XRD diffraction pattern, we believe that it should be the Al_2_Cu phase. Some α-Al+Al_2_Cu eutectic network structures are distributed along α-Al grain boundaries or between dendrites. The sub-eutectic composition of Cu determines this microstructural characteristic. In the Al-Cu alloy of this experiment, the composition of Cu was 6 wt.%, which was much lower than the eutectic point composition (33.2 wt.%). Therefore, α-Al grains became the primary phases. With the continuous growth of the α-Al grains, aluminium atoms were constantly consumed, and Cu atoms were continuously enriched to the front of the solidification interface. When the concentration of Cu in the residual liquid reached 33.2 wt.%, a eutectic reaction occurred as follows: L→α-Al+Al_2_Cu; therefore, the eutectic network structure of both was distributed at the grain boundaries or interdendritic zone.

Further observation showed some light gray phases (spot 3), and some long strips (spot 6) were observed within part of the bright white phase. According to the EDS analysis, both phases contained large amounts of Al, Cu, Mn, and Fe elements. In the light gray phase (Spot 3), the content of Al was 74.9 at.%, the content of Cu was 18.2 at.%, and the total content of Mn and Fe was 6.67at.%. The peak of the Al_7_Cu_2_Fe phase appeared in the XRD diffraction pattern, and combined with the reference [47], we believe that this phase was the Al_7_Cu_2_Fe phase. In other elongated phases (spot 6), the content of Al was 72.71 at.%, the content of Cu was 11.22 at.%, and the total content of Mn and Fe was 15.94 at.%. The peak of the Al_20_Cu_2_Mn_3_ phase appeared in the XRD diffraction pattern, and combined with the reference [48], we believe that this phase was the Al_20_Cu_2_Mn_3_ phase. According to a previous study [49], this was due to the phenomenon of mutual substitution between Fe and Mn atoms, resulting in the solid solution of the Fe and Mn elements into the original Al_7_Cu_2_Fe and Al_20_Cu_2_Mn_3_ phases, respectively, forming Al_7_Cu_2_(Fe, Mn) and Al_20_Cu_2_(Mn, Fe)_3_ phases. It seems that there was no significant change in the size and quantity of the α-Al grains and Al+Al_2_Cu eutectic network structure as the Mn/Ag ratio increased.

### 3.2. Tensile Properties of as-Cast Al-Cu-xMn-yAg Alloys

**Figure 5:**
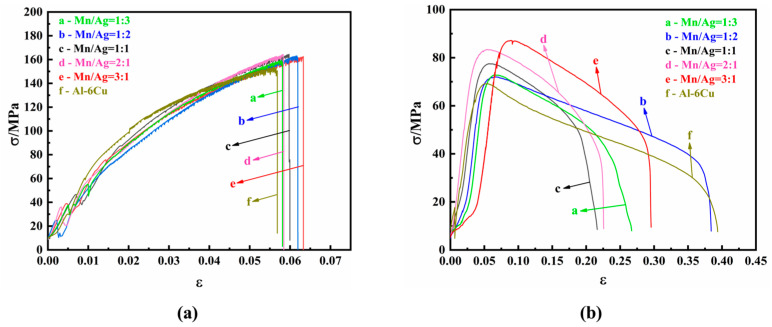
Strain–stress curves of as-cast Al-6Cu-xMn-yAg alloys: (**a**) room temperature; (**b**) 350 °C.

The mechanical properties of the as-cast alloys were studied by tensile tests at room temperature and a high temperature (350 °C). The tensile strain–stress curves are shown in Figure 5, and the specific experimental data are shown in Table 2 and Table 3.

It can be seen from Table 2 and Figure 5a that the as-cast alloy had no noticeable difference in tensile properties at room temperature. When the Mn/Ag ratio was 2:1, the as-cast alloy had the best mechanical properties; its tensile strength was 164.34 MPa, and its elongation was 2.94%. The results showed that the Mn/Ag ratio change did not affect the tensile properties of the as-cast alloys at room temperature, which was consistent with the fact that the change in the Mg/Ag ratio had a relatively small impact on the microstructure of the as-cast alloy.

It can be seen from Table 3 and Figure 5b that the tensile properties of the as-cast alloy at a high temperature (350 °C) were significantly different from those at room temperature. With a decrease of the Mn/Ag ratio, the tensile strength of the cast alloy gradually decreased, and the elongation decreased first and then increased. When the Mn/Ag ratio was 3:1, the as-cast alloy had the best tensile strength, reaching 87.17 MPa, and the elongation after fracture was 25%. The above results show that Mn was dominant in strengthening the as-cast alloy at 350 °C.

According to the literature [50], the bond strength is mainly controlled by the valence electrons of each atom. The more bonding electrons, the more muscular the bonding strength of the crystal structure, which is conducive to improving the high-temperature thermal strength; additionally, the atomic radii of Al, Cu, and Mn are 143.2 pm, 125 pm, and 139 pm [51], respectively. Therefore, when Mn atoms replace some Cu atoms, specific geometric distortion will be caused, significantly affecting the redistribution and energetics of valence electrons [52]. Mn belongs to the transitional family of elements and has an incomplete outer electron shell. After Mn atoms enter the lattice, some high-priced Mn atoms (+2~7) will replace low-priced Cu atoms (+1~2), causing local geometric distortion and losing more valence electrons. We infer that this will decrease local valence electron density and increase charge transfer, thus strengthening the interaction between Mn and the surrounding atoms. Thus, the structural stability of the Al_2_Cu phase at high temperatures can be effectively improved. Although Ag atoms also appear in the Al_2_Cu phase, like Mn atoms, the thermal stability of the Al_2_Cu phase is limited due to the lower valence state of Ag atoms (+1). Therefore, this explains why the mechanical properties of the alloy increase with an increase in Mn content at 350 °C.

**Figure 6:**
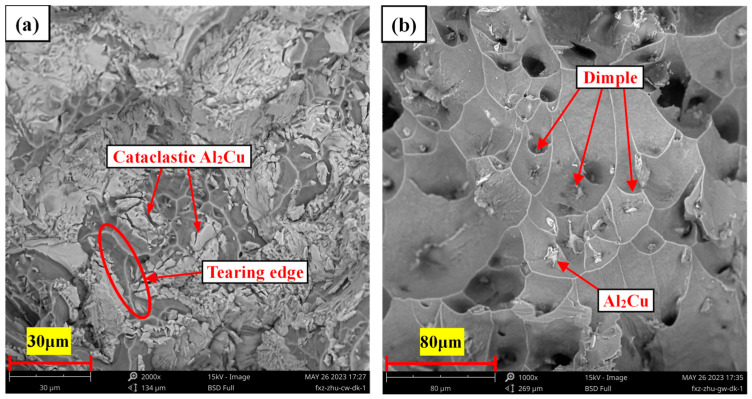
Tensile fracture morphology of as-cast Al-6Cu-0.4Mn-0.4Ag alloys: (**a**) room temperature; (**b**) 350 °C.

Figure 6 shows the room temperature and high temperature (350 °C) tensile fracture morphology of the as-cast Al-6Cu-0.4Mn-0.4Ag alloy. It can be seen from Figure 6a that the fracture surface had some shallow dimples and some smooth cleavage planes. The analysis showed that the fracture mode of the alloy was mainly brittle fracture, which was consistent with the lower elongations of the alloys listed in Table 2. The analysis showed that the cleavage planes and fragmented blocks were generated by the Al_2_Cu phase. The Al_2_Cu phase was a brittle intermetallic compound, and its coarse network structure led to stress concentrations that were apt to occur in this phase, ultimately resulting in cleavage planes and fragmentation, which accelerated the fracture of the alloy and could not effectively improve the tensile property of the alloy at room temperature. As can be seen from the high-temperature tensile fracture of the alloy in Figure 6b, many dimples covered the surface of the tensile fracture. These thick and deep dimples indicated that the alloy underwent large plastic deformation before fracture, and dislocation slip and movement were apparent. The plasticity of the alloy was considerable because the matrix softens at high temperatures, reducing sensitivity to interface cracks, which could passivate the tip of the interface cracks, delay crack propagation, and enable Al_2_Cu particles to play a precipitation-strengthening role resulting in no rapid fracture failure in the tensile process. At the bottom of the dimple were some white Al_2_Cu phase deposits, which indicated that the Al_2_Cu particles had a good strengthening effect.

### 3.3. Microstructure of the Heat-Treated Alloy

**Figure 7:**
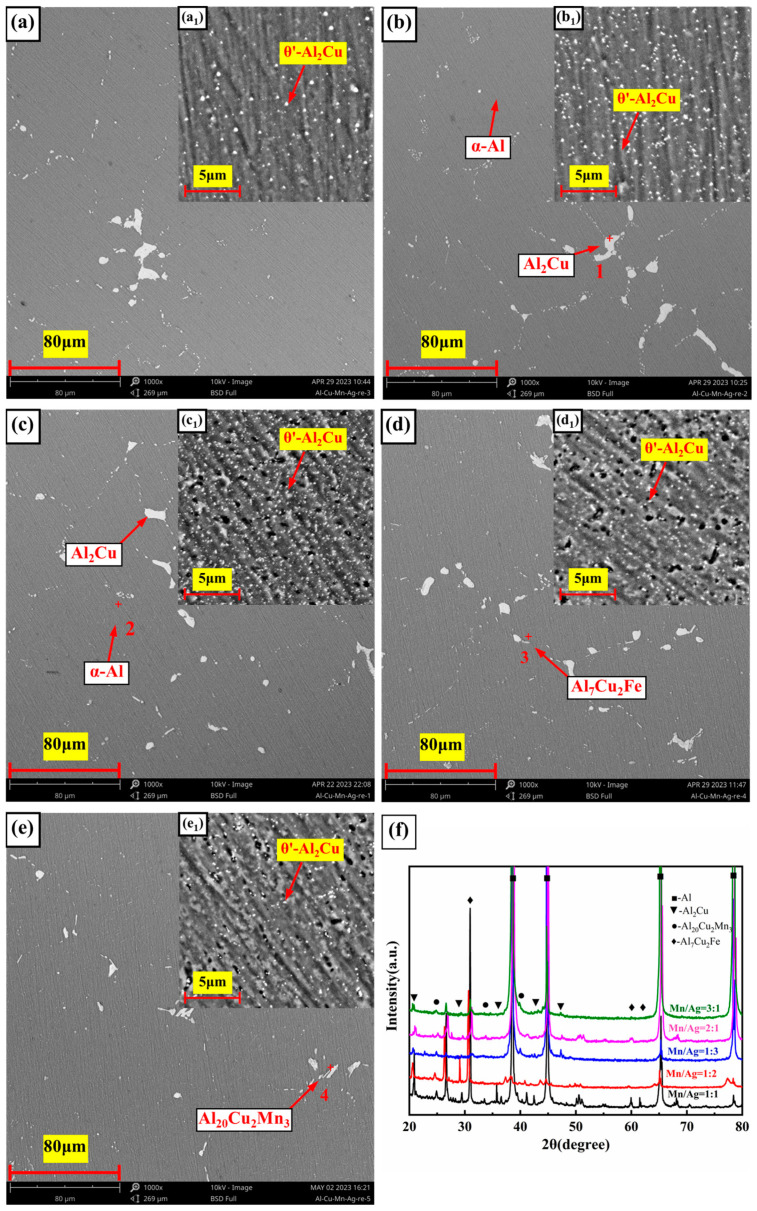
Microstructure of the heat-treated Al-6Cu-xMn-yAg alloys: (**a**,**a_1_**) x/y = 1:3; (**b**,**b_1_**) x/y = 1:2; (**c**,**c_1_**) x/y = 1:1; (**d**,**d_1_**) x/y = 2:1; (**e**,**e_1_**) x/y = 3:1; (**f**) XRD patterns of the heat-treated alloys.

**Figure 8:**
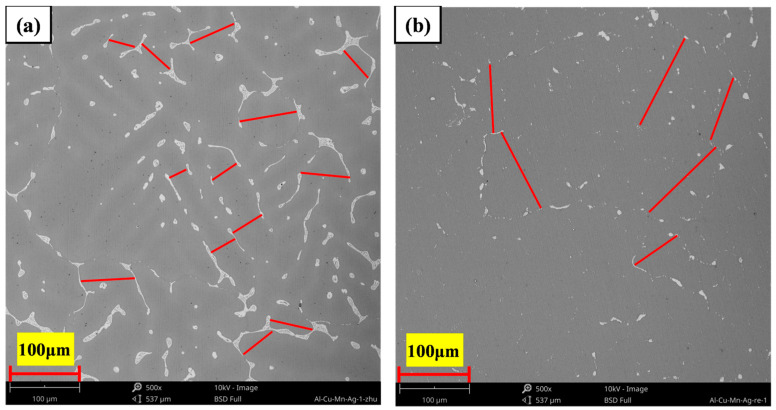
Microstructure of the Al-6Cu-0.4Mn-0.4Ag alloy: (**a**) as-cast alloy; (**b**) T6 heat-treated alloy.

**Figure 9:**
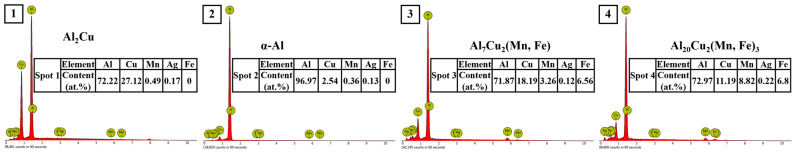
Energy spectrogram of the composition at each point in Figure 8.

Figure 7 shows the microstructure and XRD patterns of the heat-treated alloys with different Mn/Ag ratios. Figure 7a–d,e are the low-magnification images, and Figure 7a_1_–d_1_,e_1_ are the high-magnification images of the samples after metallographic corrosion. Figure 8 shows the microstructure of the as-cast and T6 heat-treated Al-6Cu-0.4Mn-0.4Ag alloys. The EDS analysis results of the chemical composition of each phase are shown in Figure 9. It can be seen from the microstructure of the SEM photos in Figure 7 and the EDS components of each phase in Figure 9 that the heat-treated alloy was mainly composed of α-Al, θ′-Al_2_Cu, Al_20_Cu_2_(Mn, Fe)_3_, and a small amount of Al_7_Cu_2_(Fe, Mn) phases.

During the heat treatment process, the alloys are heated to more than 500 °C, and most of the cast network θ-Al_2_Cu will decompose and dissolve, and Cu solute atoms will dissolve into the Al matrix to form a single-phase solid solution of α-Al. However, there are still some coarse Al_2_Cu phases in the aluminium matrix. We know that the limit solid solubility of Cu in aluminium is 5.65 wt.% [53], and the designed alloy has a copper content of 6%, so a small amount of the θ-Al_2_Cu phase will not dissolve into the matrix. According to Oswald’s ripening theory [54], the energy of large particles is lower than that of small particles, and large particles are more stable than small ones, so small particles in the thermodynamic system will be swallowed by large particles [55]. The residual θ-Al_2_Cu in this experiment would also grow into coarse particles through the Oswald ripening mechanism during the solution–aging process. As shown in Figure 7a–e, the remaining Al_2_Cu particles were relatively large.

After aging treatment, fine dispersed θ′-Al_2_Cu particles (100–400 nm) were precipitated from the alloy matrix, as shown in the high-magnification microstructure diagram (a_1_–e_1_) in Figure 7. With the increase in Mn content in the Mn/Ag ratio, the quantity and size of θ′-Al_2_Cu particles first increased and then decreased. When the Mn/Ag ratio was 1:1 (Figure 7c), θ′-Al_2_Cu particles had the most significant number and the smallest particle size, with an average particle size of 136 nm. This showed that the Mn/Ag ratio change specifically affected the quantity and size of the precipitated phase. Comparing the lengths of the red line segments in Figure 8a,b showed that the α-Al dendrites were coarsened after heat treatment. The main reason was that intense thermal diffusion occurred during the solution treatment at more than 500 °C to make the grains merge and grow [55]. These phenomena were consistent with some previous research [56].

In the heat treatment process, Mn can promote the precipitation and refinement of the θ′-Al_2_Cu phase, which is due to the low thermal diffusion coefficient of Mn, which will block the diffusion of Al and Cu, and it is easy to form a blockage. As the number of blocked atoms increases and the aggregated atomic clusters reach a specific critical size, nucleation begins. The increase in the amount of θ′-Al_2_Cu phase precipitation represents a decrease in the size of each phase [57]. At present, although there are no studies showing that the Ag element can refine the θ′-Al_2_Cu phase by promoting the precipitation of the θ′-Al_2_Cu phase, since the Ag element can segregate to the coherent interface of the θ′-Al_2_Cu phase, forming an Ag-rich double layer, thereby reducing the interfacial energy and improving the thermal stability of the phase, at the same time, the Ag element will also segregate to approximately 40% of the semi-coherent lattice θ′/matrix interface, which can hinder the lateral growth of the θ′-Al_2_Cu phase and cooperate with the Mn element to further refine the θ′-Al_2_Cu phase [40].

### 3.4. Tensile Properties of the Heat-Treated Alloys

**Figure 10:**
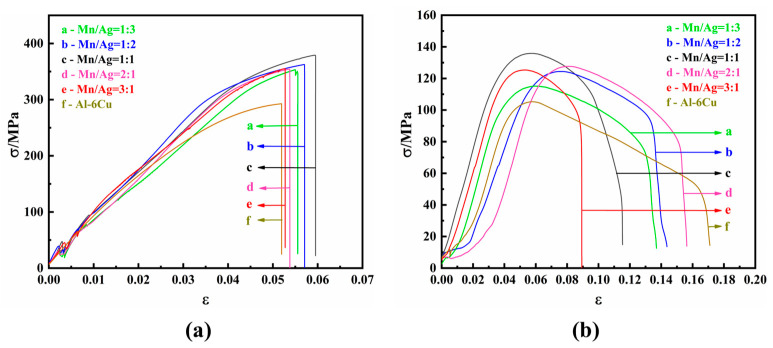
Strain–stress curves of the heat-treated Al-6Cu-xMn-yAg alloys: (**a**) room temperature; (**b**) 350 °C.

The mechanical properties of the heat-treated alloys were studied by tensile tests at room temperature and a high temperature (350 °C). The tensile strain–stress curves are shown in Figure 10, and the specific experimental data are shown in Table 4 and Table 5.

It can be seen that, at room temperature, with an increase of the Mn/Ag ratio, the tensile strength of the heat-treated alloy first increased and then decreased. When the Mn/Ag ratio was 1:1, the tensile mechanical properties of the heat-treated alloy were the best, reaching 379.14 MPa, 131.18% higher than that of the as-cast alloy, and the elongation after fracture was 2.87%. The above results showed that solution–aging heat treatment could significantly improve the mechanical properties of the alloy at room temperature.

It can be seen from Table 4 and Figure 10b that, at a high temperature (350 °C), with an increase of the Mn/Ag ratio, the tensile strength of the heat-treated alloy also first increased and then decreased, and the fracture elongation was the same. When the Mn/Ag ratio was 1:1, the tensile mechanical properties were the best, reaching 135.89 MPa, which was 75.30% higher than that of the as-cast alloy, and the elongation was 11.92%. The above results showed that heat treatment was essential in strengthening the alloy at high temperatures. Table 6 shows some heat-resistant aluminium alloys’ tensile strength at 350 °C reported in recent years. It can be seen that the maximum high-temperature tensile strength of this study exceeded that of most heat-resistant aluminium alloys reported in recent years., indicating that it has potential high-temperature applications. The improvement of tensile properties at room temperature and high temperatures after heat treatment was mainly attributed to the dispersion strengthening of the fine dispersed θ′-Al_2_Cu nanophase. The Orowan strengthening mechanism could explain this.

Orowan strengthening describes the strengthening effect resulting from the interaction between the reinforced particles and the dislocation motion in the matrix; that is, the second phase of dispersion in the matrix impedes the dislocation motion, thereby improving the material’s mechanical properties [58]. The Orowan bypass stress Δ*σ_Orowan_* is expressed as follows [59]:

(1) $${\Delta \sigma }_{Orwan}=\frac{0.4{\mathrm{MG}}_{m}b^{\ln\left(\frac{\sqrt{\left(\frac{2}{3}\right)}d_{p}}{b}\right)}}{\pi \lambda \sqrt{\left(1-V\right)}}$$

(2) $$\lambda =\sqrt{\left(\frac{2}{3}\right)}d_{p}\left(\sqrt{\left(\frac{\pi }{4V_{P}}\right)}-1\right)$$

where M is the average orientation factor (*M_Al_* = 3.06), λ is the spacing between particles, *d_p_* is the average particle size, *b* is the Burgers vector, *V_P_* is the volume fraction of strengthened particles, *G_m_* is the shear modulus of the matrix, and ν is the Poisson’s ratio. Orowan strengthening is usually initiated by particles smaller than 1 μm in size, and the θ′ phase in the heat-treated alloy in this study was between 0.1 and 0.4 μm in size. In this experiment, when the Mn/Ag ratio was 1:1, the θ′-Al_2_Cu precipitates were the of an enormous amount and the smallest size, so the highest tensile mechanical properties were obtained, which accorded with the Orowan strengthening mechanism.

**Table 6:** Comparison of tensile strength with other heat-resistant aluminum alloys at 350 °C.

Materials Composition (wt.%)	Temperature (°C)	σUTS (MPa)	Year	Ref.
Al-6Cu-0.4Mn-0.4Ag	350	135.89	2024	Present work
Al-5Cu-1.5Ni-0V	350	92.06	2023	[55]
Al-5Cu-1.5Ni-0.3V	350	111.81	2023	[55]
Al-6.5Cu-2Ni-0.5Zr-0.3Ti-0.25V	350	127.5	2023	[60]
Al-6Cu-2Ni-0.5V	350	123.4	2023	[57]
9 wt.%Al_3_Zr/Al-6Cu-2Ni-0.5V	350	136.9	2023	[57]
Al-11.93Si-4.03Cu-0.97Mg-2.67Ni-0.52Fe	350	103	2022	[61]
rGO/Al	350	128	2020	[62]
(2% Al3Zr + 15.2% Al3Ni)/Al-1Mg-0.8Mn-0.8V	350	82	2020	[63]
Al-12.16Si-3.95Cu-1.01Mg-2.54Ni-0.47Fe	350	100	2019	[64]
Al-11.98Si-4.02Cu-1.02Mg-2.68Ni-0.62Fe	350	106	2019	[64]
Al-12Si-4Cu-2Ni-1Mg-AlNp	350	106	2019	[6]
Al-12.95Si-3.57Cu-0.72Mg-0.91Ni-0.53Fe-0.4Er	350	117	2019	[65]

**Figure 11:**
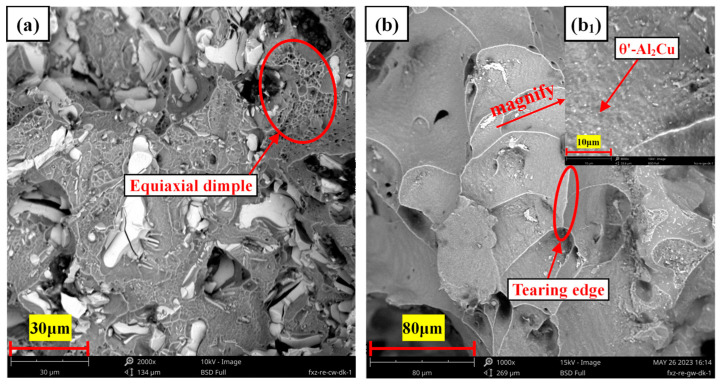
Tensile fracture morphology of the heat-treated Al-6Cu-0.4Mn-0.4Ag alloys: (**a**) room temperature; (**b**,**b_1_**) 350 °C.

Figure 11 shows the room temperature and high temperature (350 °C) tensile fracture morphology of the heat-treated Al-6Cu-0.4Mn-0.4Ag alloy. It can be seen that the room temperature tensile fracture surface of the alloy was mainly composed of some white cleavage planes and some small dimples. Many massive Al_2_Cu phases were distributed in the fracture, which was caused by the failure of the mid-layer sheet θ-Al_2_Cu phase to dissolve entirely during the solid solution process. During the tensile process, these phases produced stress concentration and became the crack source, thus accelerating the fracture failure of the matrix alloy. Compared with the as-cast alloy, a large amount of the θ′-Al_2_Cu phase was precipitated in the microstructure of the heat-treated alloy. The precipitation size of the θ′ phase was small, which made it difficult for the dislocation to the shear θ′ phase. Only when the external stress reached a certain degree could the θ′ phase be bypassed to deflect the α-Al phase along the surface of θ′-Al_2_Cu, which could effectively inhibit crack growth and significantly affect the mechanical properties of the matrix alloy. It can be seen from the high-temperature tensile fracture of the heat-treated alloys in Figure 11b that the high-temperature fracture of the T6 alloy was mainly composed of deep and wide dimples, and the high-temperature tensile fracture mode of the alloy belonged to ductile fracture. After heat treatment, more θ′-Al_2_Cu nanophases were precipitated (Figure 11b_1_). It can be seen that many fine θ′-Al_2_Cu phases were precipitated on the inner wall of the dimple. However, no small dimple was generated, indicating that the interface of the θ′-Al_2_Cu nanophase and matrix was firmly bonded. It could play a good role in preventing the slip of matrix dislocation. θ′-Al_2_Cu particles prevented the dislocation movement of the matrix, resulting in a decrease in plasticity and an increase in strength.

**Figure 12:**
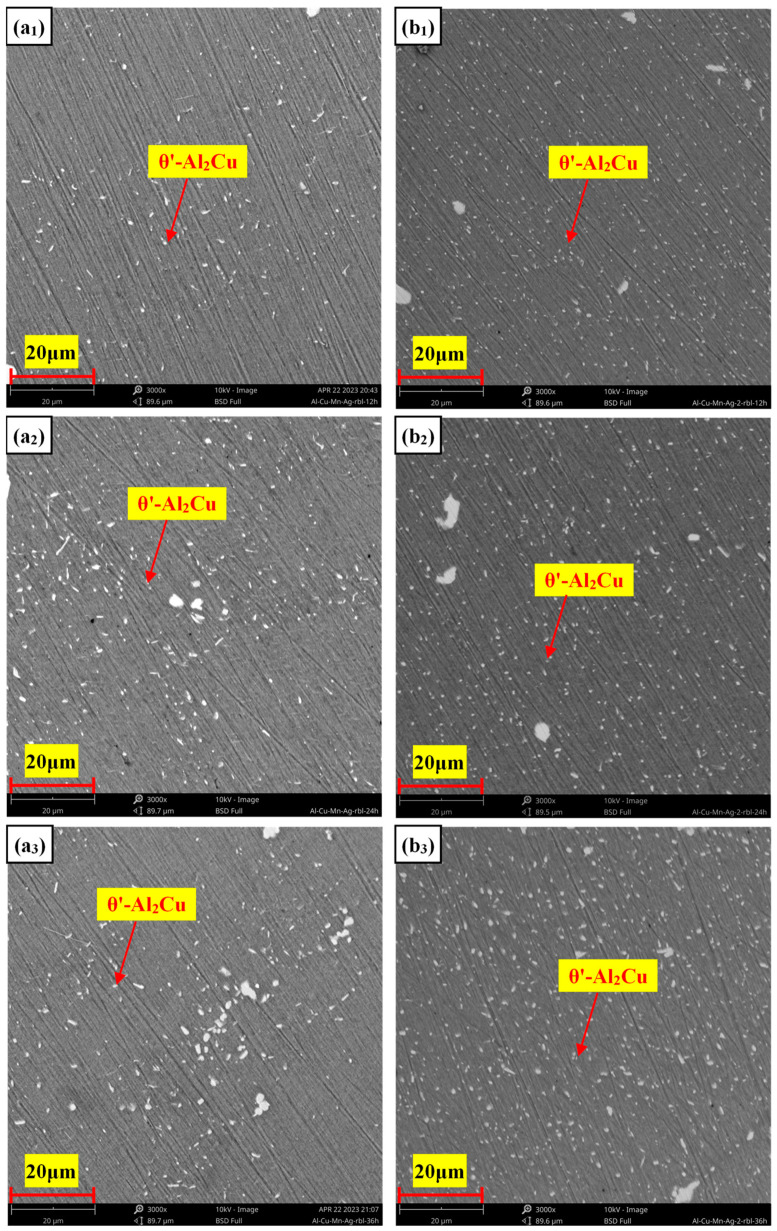
Microstructure of the Al-Cu-Mn-Ag alloys exposed to a heat of 350 °C at different times (T): Al-6Cu-0.2Mn-0.6Ag: (**a_1_**) T = 12 h; (**a_2_**) T = 24 h; (**a_3_**) T = 36 h; Al-6Cu-0.4Mn-0.4Ag: (**b_1_**) T = 12 h; (**b_2_**) T = 24 h; (**b_3_**) T = 36 h.

High-temperature mechanical properties are influenced by both the size and number of strengthening phase particles, as well as their strength and thermal stability. If the particles soften or coarsen at high temperatures, mechanical properties will significantly suffer. Recent studies have shown that adding Mn can promote the precipitation of the θ′ phase and refine the size of the θ′ during the artificial aging process. It can improve the high-temperature coarsening resistance of the θ′ phase, improving its thermal stability. The main reason is that Mn atoms segregate at the interface of θ′/α-Al, reducing the interface energy of both and thus improving the thermal stability of the θ′ phase. When Ag is added to an Al-Cu alloy, the Ag atom can form a diatomic layer at the θ′/α-Al coherent interface during the aging process, and the interface energy of the two phases can also be reduced, which promotes an increase in θ′. In this experiment, Mn and Ag were added to an Al-Cu alloy at the same time, and it was found that when Mn/Ag = 1:1, that is, the atomic ratio was about 2:1, the θ′-Al_2_Cu precipitation quantity was the largest and the size was the smallest. Therefore, we conclude that the joint segregation of Mn and Ag at the θ′/α-Al interface had a synergistic effect. The precipitation quantity and size of the θ′ phase could be minimized. Since the best high-temperature mechanical properties were also found in the Mn/Ag = 1:1 alloy, we believe that the thermal stability of the θ′ phase also plays a role. To this end, we conducted thermal exposure experiments on alloys with Mn/Ag ratios of 1:3 and 1:1 at different times to verify their anti-coarsening abilities. The result is shown in Figure 12.

**Figure 13:**
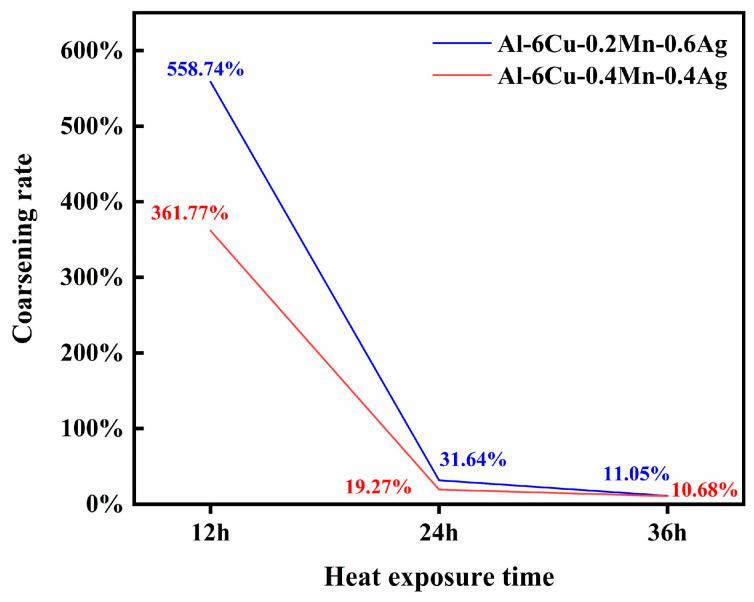
Heat exposure coarsening rate curve of Al-6Cu-0.2Mn-0.6Ag and Al-6Cu-0.4Mn-0.4Ag alloys.

It can be seen that the θ′ phase size of both alloys increased with the extension of thermal exposure time, and the θ′-Al_2_Cu phase gradually increased and grew in the longitudinal direction [25]. Overall, the θ′ phase size of the Mn/Ag = 1:1 alloy was smaller at different stages. As shown in Figure 13, its coarsening rate was also lower than that of the Mn/Ag = 1:3 alloy, so it can be confirmed that the Mn/Ag ratio had a significant effect on the anti-coarsening ability of the θ′ phase, that is, the effect of both on the thermal stability of the θ′ phase was also synergistic. This could be attributed to the segregation of Ag and Mn atoms at the Al/θ′-Al_2_Cu interface. Specifically, phase field simulations have suggested the synergistic roles of thermodynamic and kinetics effects in retarding the coarsening of θ′-Al_2_Cu precipitates [66,67,68]. In the thermodynamics aspect, the θ′-Al_2_Cu particles possess a plate-like morphology in the Al matrix with a coherent (001)_Al_||(001)_θ′_ interface along the broad faces and a semi-coherent (001)_Al_||(001)_θ′_ interface at the periphery of the plate. An atomically sharp Al/θ′-Al_2_Cu semi-coherent interface (with low interfacial energy) is favorable at 350 °C [52]. Mn and Ag show a strong segregation tendency at the Al/θ′-Al_2_Cu interface, which provides powerful chemical bonds. Solute segregation can compensate for a lattice mismatch at the semi-coherent interface between Al and θ′-Al_2_Cu; the chemical interaction of solutes at the interface helps reduce the interface energy [14]. In the kinetics aspect, the thermal diffusion coefficient of the Mn atom is much lower than that of other atoms, which means that if it were introduced into the alloys, it would diffuse very slowly at high temperatures and could hinder the diffusion of other atoms, which is what we would expect, because it serves as a “coarsening barrier” and helps to hinder the suppression of the freely available vacancies in the Al matrix for Cu jumping (which leads to the coarsening of θ′-Al_2_Cu) [66,69]. According to some reports [25], it can be supposed that in the early stage of thermal exposure, Ag atoms with a relatively high thermal diffusion coefficient may first reach the Al/θ′-Al_2_Cu interface and control the structure coarsening of θ′-Al_2_Cu within a reasonable range. Subsequently, the Mn atoms with a lower thermal diffusion coefficient reach the interface to prevent the formation of a Cu surface on the interface and prevent the interface from becoming thicker and coarser.

## 4. Conclusions

This study investigated the effects of the Mn/Ag ratio and heat treatment microalloying on the microstructures and mechanical properties of Al-6Cu-xMn-yAg alloys. The main conclusions are summarized as follows:

(1) The as-cast and heat-treated Al-Cu-Mn-Ag alloys mainly comprised α-Al and Al2Cu phases. In the as-cast alloy, the Mn/Ag ratio had no noticeable effect on the microstructure of the alloy, and the reticular (θ′-Al_2_Cu+α-Al) eutectic structure was distributed along the grain boundaries of the primary α-Al grains in the as-cast alloy. After T6 heat treatment, the α-Al dendrite phase was coarsened. The fine nanocrystalline θ′-Al_2_Cu particles were precipitated from the matrix. With the Mn/Ag ratio increase, precipitation and particle size increased first and then decreased. When the Mn/Ag ratio was 1:1, the precipitated quantity of the θ′-Al_2_Cu phase reached the highest and the smallest size.
(2) At room temperature, the ultimate tensile strength of the as-cast alloy did not significantly change, and the average tensile strength was about 163.5 MPa. At high temperatures, the ultimate tensile strength of the as-cast alloy increased with the increase of the Mn/Ag ratio, and the highest tensile strength was 87.17 MPa. After heat treatment, the alloy’s ultimate tensile strength and yield strength at room temperature and high temperatures were significantly increased. As the Mn/Ag ratio increased, it increased and then decreased. When the Mn/Ag ratio was 1:1, the ultimate tensile strength at room temperature and high temperatures reached the highest, which were 379.14 MPa and 135.89 MPa, respectively.
(3) In the heat treatment process of the alloy, due to the low thermal diffusion coefficient of Mn, the diffusion of Al and Cu could be blocked to form a blockage of atoms and then promote the nucleation and refinement of the θ′-Al_2_Cu phase, while the Ag element could also cooperate with the Mn element to further the fine θ′-Al_2_Cu phase and promote its thermal stability. Through the optimization of the Mn/Ag ratio, we found that when the Mn/Ag ratio was 1:1, the θ′-Al_2_Cu phase had the best thermal stability, which indicated that the interface energy of the θ′-Al_2_Cu phase of this ratio alloy was the lowest, which was the most favorable for anti-coursing, so its high-temperature mechanical properties were the highest.

## Figures and Tables

**Table 1:** Nominal composition of the studied alloys (wt.%).

Alloys	Mass Fraction/%				Mn/Ag
No.	Cu	Mn	Ag	Al	Weight Ratio
#1	6	0.2	0.6	Bal.	1:3
#2	6	0.27	0.53	Bal.	1:2
#3	6	0.4	0.4	Bal.	1:1
#4	6	0.53	0.27	Bal.	2:1
#5	6	0.6	0.2	Bal.	3:1
Al-6Cu	6	-	-	Bal.	-

**Table 2:** Mechanical property data of as-cast Al-6Cu-xMn-yAg alloys tested at room temperature (27 °C).

Alloys	Mn/Ag	UTS(σb)/MPa	YS(σs)/MPa	FS/%	El./%
#1	1:3	163.19	156.99	6.20	3.2
#2	1:2	162.53	156.65	6.33	3.31
#3	1:1	164.00	159.65	5.99	3.18
#4	2:1	164.34	159.63	5.85	2.94
#5	3:1	163.68	151.40	5.81	3.13
Al-6Cu	-	153.55	143.38	6.82	3.36

**Table 3:** Mechanical property data of as-cast Al-6Cu-xMn-yAg alloys tested at 350 °C.

Alloys	Mn/Ag	UTS(σb)/MPa	YS(σs)/MPa	FS/%	El./%
#1	1:3	72.90	72.06	26.69	22.87
#2	1:2	72.02	65.04	38.40	35.43
#3	1:1	77.52	73.71	22.16	18.85
#4	2:1	83.40	77.21	23.57	19.60
#5	3:1	87.17	86.46	29.56	25.00
Al-6Cu	-	69.22	60.36	39.37	35.88

**Table 4:** Mechanical property data of the heat-treated Al-6Cu-xMn-yAg alloys tested at room temperature (27 °C).

Alloys	Mn/Ag	UTS(σb)/MPa	YS(σs)/MPa	FS/%	El./%
#1	1:3	353.28	328.69	5.55	2.63
#2	1:2	362.56	336.72	5.71	2.72
#3	1:1	379.14	345.73	5.95	2.87
#4	2:1	354.29	329.73	5.38	2.58
#5	3:1	355.29	328.83	5.27	2.43
Al-6Cu	-	292.11	261.16	5.19	2.07

**Table 5:** Mechanical property data of the heat-treated Al-6Cu-xMn-yAg alloys tested at 350 °C.

Alloys	Mn/Ag	UTS(σb)/MPa	YS(σs)/MPa	FS/%	El./%
#1	1:3	115.15	104.21	13.69	10.90
#2	1:2	124.42	118.31	14.37	11.02
#3	1:1	135.89	122.26	14.83	11.92
#4	2:1	127.69	115.75	15.63	12.55
#5	3:1	125.37	114.23	8.94	7.05
Al-6Cu	-	105.39	93.21	17.09	14.33

## Data Availability

Data are contained within the article.
